# Reducing tectorial membrane viscoelasticity enhances spontaneous otoacoustic emissions and compromises the detection of low level sound

**DOI:** 10.1038/s41598-019-43970-5

**Published:** 2019-05-16

**Authors:** Thomas Bowling, Charlsie Lemons, Julien Meaud

**Affiliations:** 1 G.W.W. School of Mechanical Engineering, Georgia Institute of Technology, 771 Ferst Drive NW, Atlanta, Georgia 30332 USA; 2 Petit Institute for Bioengineering and Bioscience, Georgia Institute of Technology, Atlanta, GA 30332 USA

**Keywords:** Computational biophysics, Cochlea

## Abstract

The mammalian cochlea is able to detect faint sounds due to the presence of an active nonlinear feedback mechanism that boosts cochlear vibrations of low amplitude. Because of this feedback, self-sustained oscillations called spontaneous otoacoustic emissions (SOAEs) can often be measured in the ear canal. Recent experiments in genetically modified mice have demonstrated that mutations of the genes expressed in the tectorial membrane (TM), an extracellular matrix located in the cochlea, can significantly enhance the generation of SOAEs. Multiple untested mechanisms have been proposed to explain these unexpected results. In this work, a physiologically motivated computational model of a mammalian species commonly studied in auditory research, the gerbil, is used to demonstrate that altering the viscoelastic properties of the TM tends to affect the linear stability of the cochlea, SOAE generation and the cochlear response to low amplitude stimuli. These results suggest that changes in TM properties might be the underlying cause for SOAE enhancement in some mutant mice. Furthermore, these theoretical findings imply that the TM contributes to keeping the mammalian cochlea near an oscillatory instability, which promotes high sensitivity and the detection of low level stimuli.

## Introduction

The mammalian cochlea is a remarkable sensory system with extremely high sensitivity, sharp frequency selectivity and broad dynamic range. Such impressive characteristics are products of an active feedback mechanism commonly called the cochlear amplifier^1^. Because of the cochlear amplifier, the cochlea is an active system that operates close to a dynamic instability at low levels that are close to the hearing threshold^2,3^. The cochlea is sometimes in an oscillatory regime characterized by the presence of self-sustained oscillations, as predicted by Gold in 1948^2^ and observed more than 30 years later by recording spontaneous oscillations in the ear canal pressure^4^. These narrow-band oscillations, called spontaneous otoacoustic emissions (SOAEs), are quite common in humans and can be recorded in about 70% of the population^5^. While they do not play any functional role, SOAEs are a signature of the cochlear amplifier and provide insight into the fundamental biophysics of the cochlea at low levels.

In mammalian species, two different active cellular processes located in outer hair cells (OHCs) might play a role in cochlear amplification and SOAE generation: piezoelectric-like somatic electromotility of the OHC main body^6^ and hair bundle (HB) motility^7^, *i.e*., motility of the hair-like filaments located on top of the OHCs. Somatic electromotility is known to be essential for mammalian cochlear amplification^1,8^; additionally, experiments and theoretical models^9–12^ have shown that HB motility might also be an important component of the cochlear amplifier. Because spontaneous oscillations of HBs have been observed in non-mammalian vertebrates^13,14^, HB motility has often been hypothesized to underlie the generation of SOAEs in these species. However, spontaneous oscillations of mammalian HBs have never been observed, even though some evidence of mammalian HB motility has been observed^15,16^. Due to the inherent coupling between OHCs linked to the presence of a traveling wave and of structural coupling^17,18^, mammalian SOAEs might not be the result of activity of individual HBs or OHCs but rather a global phenomenon that emerges from the active dynamics of the overall system^3,19^. In a well-established theory^19^, the cochlea acts like a laser cavity; SOAE generation requires coherent reflection of traveling waves by putative inhomogeneities in the cochlear partition (caused, for example, by cellular disorganization) and amplification of waves by OHCs. While alternative theoretical models exist^20,21^, this theory has been implemented in physically-motivated cochlear models^22^ and successfully captures many of the key characteristics of SOAEs, such as the presence of discrete spectral peaks with a common spacing between adjacent SOAEs^22,23^.

In response to an acoustic stimulus, a traveling wave propagates on the basilar membrane (BM), the main structural component of the mammalian cochlear partition^24^. The tectorial membrane (TM) is an extracellular matrix that is located directly above the OHCs and whose role in cochlear amplification is actively debated^25–30^. The TM contains collagen fibrils embedded in a striated sheet matrix^31,32^ that consists of non-collageneous proteins, including α-tectorin (TECTA), *β*-tectorin (TECTB) and CEACAM16. Mutations of the genes that encode the TM proteins affect important characteristics of cochlear function, such as the sensitivity^33,34^ and tuning^35^ of the cochlear response to sound. Interestingly, recent experiments in transgenic mice have also shown that altering the properties, structure and morphology of the TM tends to enhance the generation of SOAEs^36–38^. This could imply that the TM helps maintain cochlear stability and prevent the generation of too many spontaneous oscillations. Beyond its fundamental scientific significance, studying the effect of TM genetic mutations could have clinical implications, since several TM mutations have been linked to hereditary hearing disorders in humans: for example, the *Tecta*^*Y*1870/+^ and *Ceacam16* knock-out (KO) mice are models for human deafness DFNA8^39^ and DFNA4B^40^, respectively.

However, TM mutations typically have multiple effects on the morphology and properties of the TM, making it difficult to link individual changes in TM properties to the observed changes in cochlear physiology using an approach based only on experiments. For example, multiple TM mutations are known to affect its mechanical properties: both the *Tectb* KO and the mutations tend to reduce the shear stiffness of the TM^41–43^ but only the TMs have lower shear viscosity than the wild-type (WT) TMs^42^. Additionally, a loss of the Hensen’s stripe^35^ (a narrow ridge normally located on the bottom surface of the TM) is observed in *Tectb* KO mice; the presence of holes are observed in the TM of *Ceacam16* KO^36^ and *Tecta*^*Y*1870/+^ mice^44^.

Theoretical results^18^, along with analysis of the relationship between cochlear tuning and the spatial extent of longitudinal TM waves^42^, suggest that TM properties, and particularly the viscosity of the TM, influence the sharpness of cochlear tuning. Because tuning and linear stability are tightly linked in a dynamical system, our hypothesis is that changes in the TM mechanical properties enhance the generation of SOAEs. In this paper, a physiologically-motivated computational model of the gerbil cochlea is used to examine this hypothesis.

### Overview of the model of the gerbil cochlea

**Figure 1:**
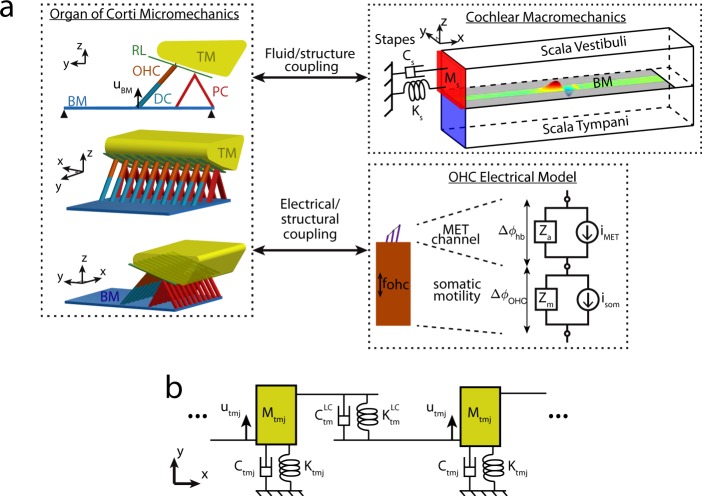
(**a**) Schematic of the 3D cochlear model. Acoustic pressure in the cochlear ducts excites the BM. The BM is coupled to a micromechanical model of the organ of Corti. In the schematic of the micromechanical model, the spacing between cross sections has been exaggerated for visualization purposes. Only one OHC is shown per cross section because the model lumps the three OHCs into one. The Deiter’s cell (DC) and the pillar cells (PC) are modeled as rigid. The cross-sections of the reticular lamina (RL) are not directly coupled, while longitudinal coupling is included for the BM and TM. This micromechanical model is coupled to an electrical model of the OHC. Stimulation of the OHC HB generates a mechano-electrical transduction (MET) current, *i*_*MET*_ (Eq. 4). This current depolarizes the OHCs (Eq. 8), which generate an active force, *f*_*ohc*_ (Eq. 6), due to electromotility. The cochlear model is coupled to a one DOF middle ear model. (**b**) Model of the TM with TM viscoelastic longitudinal coupling. *x* corresponds to the longitudinal direction. ${K}_{tm}^{LC}$ and ${C}_{tm}^{LC}$ correspond to the longitudinal coupling stiffness and viscosity of the TM, respectively; *K*_*tmj*_ and *C*_*tmj*_ are the stiffness and viscosity of the attachment of the TM to the spiral limbus, respectively in shear (*j* = *s*) and bending (*j* = *b*) modes (Eq. 10).

The finite element model used in this study is based on previously developed cochlear models^18,45–47^. This model couples an incompressible and inviscid acoustic fluid (modeled in three dimensions) with the micromechanics of the organ of Corti and the nonlinear biophysics of the OHC, as shown in Fig. 1. The OHC model takes into account somatic electromotility and nonlinear HB mechano-electrical transduction, as described in the Methods. The organ of Corti model includes degrees of freedom (DOFs) for the BM and the TM. A unique feature of this model is that the governing equation for the TM includes elastic and viscous longitudinal coupling terms that are proportional to the shear modulus and viscosity of the TM, respectively (see Fig. 1b). To test our hypothesis, four different models were considered: (1) the baseline model; (2) the “Reduced elastic” model (where the TM coupling stiffness, ${K}_{tm}^{LC}$, is reduced to 50% of its baseline value); (3) the “Reduced viscous” model (where the TM coupling viscosity, ${C}_{tm}^{LC}$, is reduced to 50% of its baseline value); (4) the “Reduced both” model (where the stiffness and viscosity are reduced to the values of the “Reduced elastic” and “Reduced viscous” models, respectively). These changes in the TM properties are representative of the changes in the TM shear modulus and viscosity reported for *Tecta*^*Y*1870/+^ mice^42^.

The cochlear model was calibrated by comparison to *in vivo* BM measurements at the base of gerbil cochlea (Supplementary Fig. S1). As seen in Supplementary Fig. S2, the model predicts an active and nonlinear response at the base of cochlea, as observed in experiments: for locations with characteristic frequencies between ∼6 and ∼40 kHz, OHCs boost the BM response to low amplitude sound by at least 20 dB. However, the effect of OHCs is much more limited at more apical locations. Thus, the validity of the model is restricted to basal locations.

Because the model does not predict any SOAE if the parameters vary smoothly, cochlear roughness was introduced by adding random perturbations (of amplitude less than 1%) to the value of a key parameter that characterizes the property of OHC somatic electromotility, the OHC electromechanical coupling coefficient, *ε*_3_ (Eqs 6 and 12). These random perturbations represent the effect of cellular disorganization and inhomogeneities in the number and properties of OHCs^48^. Because of the randomness, a random seed (RS) is chosen to initialize a random number generator. Simulating the model with different RS mimics the measurements of SOAEs in different individual cochleae within a population.

## Results

### Effect of reducing TM longitudinal coupling on SOAE generation for one seed number

**Figure 2:**
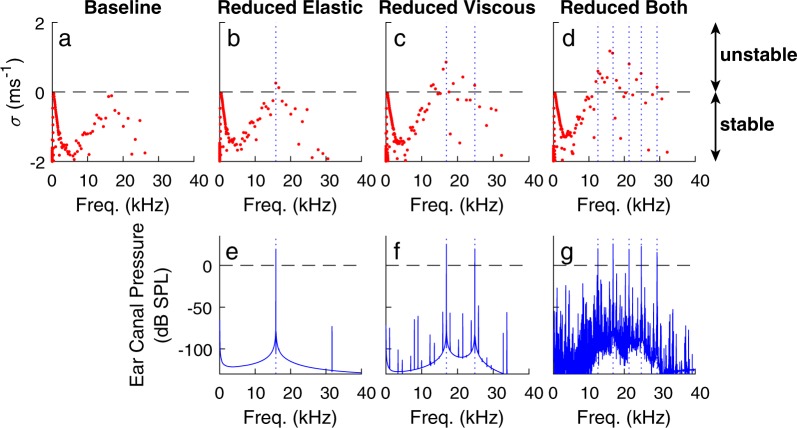
Influence of TM viscoelastic coupling on the linear stability diagram and SOAEs for one random seed (RS = 3). Linear stability diagrams (**a**–**d**) and spectrum of the ear canal pressure (**e**–**g**) for models with baseline TM coupling (**a**), reduced elastic coupling (**b**,**e**), reduced viscous coupling (**c**,**f**), and reduced elastic and viscous coupling (**d**,**g**). Δ*R* was set to 0.75% for these results. The vertical dotted lines in (**e**–**g**) represent the frequencies of spectral peaks above 0 dB SPL.

The influence of TM viscoelastic coupling on SOAE generation was first determined for one RS (Fig. 2). While experiments with transgenic animals only allow for the characterization of the influence of TM properties on a population of animals, this model-based approach makes it possible to determine how varying TM properties affects individual SOAEs.

Because SOAEs are generated when the cochlea is linearly unstable^49^, it is useful to first examine the poles, *λ*, of the model, which can be written in the following form:

1 $$\lambda =\sigma +i2\pi F$$

where *i* is the unit imaginary number, *σ* is the real part and 2*πF* is the imaginary part. A positive value for *σ* indicates that the model has a linearly unstable mode that grows exponentially and oscillates at the frequency *F*^49^. The baseline model parameters were chosen so that the model does not have any unstable modes but is just on the verge on instability for the RS used in Fig. 2, as evidenced by the presence of two nearly unstable modes (*i.e*, poles that are on the stable side and very close to the horizontal axis), of frequencies *F* ≈ 15.9 and 16.8 kHz. These nearly-unstable modes become unstable in the “Reduced elastic” model (with almost the same frequency, *F* ≈ 15.8 and 16.7 kHz). Reducing TM viscous coupling considerably reduces the linear stability: the “Reduced viscous” and “Reduced both” models have eight and fourteen linearly unstable modes, respectively. The linearly unstable modes seen in the “Reduced viscous” and “Reduced both” models (Fig. 2c,d) are either unstable or nearly unstable modes in the baseline and “Reduced elastic” models (Fig. 2a,b).

The actual cochlea emits SOAEs in the absence of any stimulus due to the presence of biological and thermal noise that causes the cochlea to move away from any unstable equilibrium. While noise could potentially be added at the stapes or in the cochlea (for example in the mechano-electrical transduction channels), we chose, as in previous work^22,50^, to apply a stimulus (an acoustic click of short duration) at the stapes instead of directly modeling noise to move the model from its unstable equilibrium configuration. While the ear canal pressure decays to 0 if the model is linearly stable, it grows until limit cycle oscillations develop due to the saturation of the mechanoelectrical transduction channels when linearly unstable modes are present. As in experiments^36^, these limit cycle oscillations appear as narrow-band spectral peaks in the ear canal pressure and correspond to SOAEs. Only spectral peaks of amplitude >0 dB SPL were counted as SOAEs in the model analysis since low amplitude peaks would be below the noise floor in an experiment. For the RS used in Fig. 2, no SOAEs are predicted by the baseline model due to a lack of linearly unstable modes. The “Reduced elastic”, “Reduced viscous” and “Reduced both” models predict one, two and five SOAEs, respectively (Fig. 2e–g). While the frequencies of SOAEs in Fig. 2e–g correspond to the frequencies of some of the linearly unstable modes in Fig. 2b–d, some of the linearly unstable modes do not appear as SOAEs; this is because some SOAEs dominate the overall response of the system, as discussed in^22^.

### Influence of TM longitudinal coupling on average number of SOAEs

**Figure 3:**
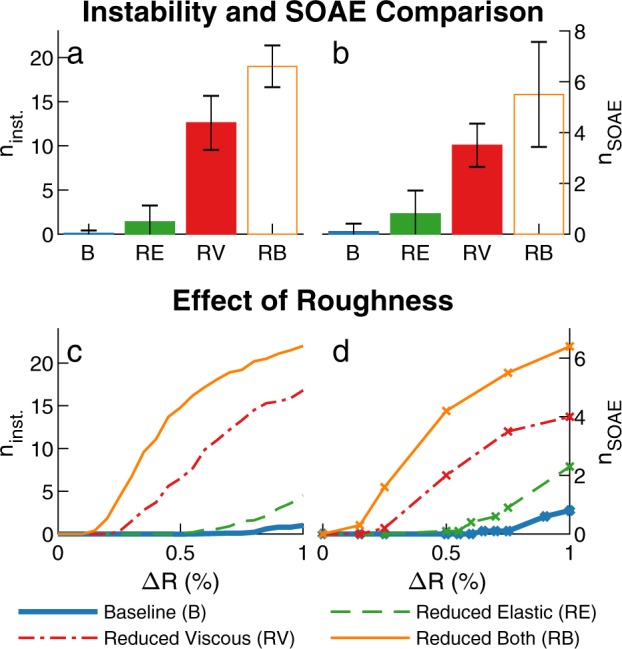
Influence of TM viscoelastic coupling on the number of linearly unstable modes and SOAEs, for Δ*R* = 0.75% and *N* = 10 different RS. (**a**,**b**) Average number of instabilities (**a**) and SOAEs (**b**) for different TM longitudinal coupling cases. The error bar corresponds to ± one standard deviation. (**c**,**d**) Average number of unstable modes (**c**) and SOAEs (**d**) as function of the amplitude of the random perturbations, Δ*R*. Supplementary Fig. S6 shows the average number of unstable modes for a wider range Δ*R* values.

The numbers of linearly unstable modes and SOAEs depend on the RS used to initialize the random number generator. The results of multiple simulations (obtained with *N* = 10 different RS) were analyzed to mimic the recording of experimental data from multiple individuals within a population of WT or mutant animals (as done for example in^36^). As seen in Fig. 3b, reducing viscoelastic coupling increases the average number of SOAEs emitted per model from 0.1 in the baseline model to 0.5 in the “Reduced elastic” model, 3.5 in the “Reduced viscous” model, and 5.5 in the “Reduced both” model. Changes in the average number of SOAEs are positively correlated with changes in the average number of linearly unstable modes (Fig. 3a). However, because one dominant mode can suppress another mode for models with multiple unstable modes (as seen in Fig. 2), the average number of unstable modes tends to be higher than the average number of SOAEs.

Because SOAE generation depends on the amplitude, Δ*R*, of the random perturbations used to introduce cochlear roughness (Eq. 12), additional simulations were examined to determine whether the conclusions regarding the influence of TM viscoelastic longitudinal coupling are robust to changes in the value of Δ*R*. As seen in Fig. 3c,d, increasing Δ*R* increases the number of linearly unstable modes and SOAEs for all TM coupling cases. However, for any given Δ*R* value, the average numbers of unstable modes and SOAEs are always ordered in the same manner:

2 $$\begin{array}{c}{n}_{inst.}^{{\rm{r}}{\rm{e}}{\rm{d}}{\rm{u}}{\rm{c}}{\rm{e}}{\rm{d}}\,{\rm{b}}{\rm{o}}{\rm{t}}{\rm{h}}}\ge {n}_{inst.}^{{\rm{r}}{\rm{e}}{\rm{d}}{\rm{u}}{\rm{c}}{\rm{e}}{\rm{d}}\,{\rm{v}}{\rm{i}}{\rm{s}}{\rm{c}}{\rm{o}}{\rm{u}}{\rm{s}}}\ge {n}_{inst.}^{{\rm{r}}{\rm{e}}{\rm{d}}{\rm{u}}{\rm{c}}{\rm{e}}{\rm{d}}\,{\rm{e}}{\rm{l}}{\rm{a}}{\rm{s}}{\rm{t}}{\rm{i}}{\rm{c}}}\ge {n}_{inst.}^{{\rm{b}}{\rm{a}}{\rm{s}}{\rm{e}}{\rm{l}}{\rm{i}}{\rm{n}}{\rm{e}}}\\ {n}_{SOAE}^{{\rm{r}}{\rm{e}}{\rm{d}}{\rm{u}}{\rm{c}}{\rm{e}}{\rm{d}}\,{\rm{b}}{\rm{o}}{\rm{t}}{\rm{h}}}\ge {n}_{SOAE}^{{\rm{r}}{\rm{e}}{\rm{d}}{\rm{u}}{\rm{c}}{\rm{e}}{\rm{d}}\,{\rm{v}}{\rm{i}}{\rm{s}}{\rm{c}}{\rm{o}}{\rm{u}}{\rm{s}}}\ge {n}_{SOAE}^{{\rm{r}}{\rm{e}}{\rm{d}}{\rm{u}}{\rm{c}}{\rm{e}}{\rm{d}}\,{\rm{e}}{\rm{l}}{\rm{a}}{\rm{s}}{\rm{t}}{\rm{i}}{\rm{c}}}\ge {n}_{SOAE}^{{\rm{b}}{\rm{a}}{\rm{s}}{\rm{e}}{\rm{l}}{\rm{i}}{\rm{n}}{\rm{e}}}\end{array}$$

where *n*_*inst*_. and *n*_*SOAE*_ denote the average number of unstable modes and SOAEs, respectively. This implies that for all Δ*R* values, reducing either elastic or viscous coupling reduces linear stability and enhances SOAE generation.

**Figure 4:**
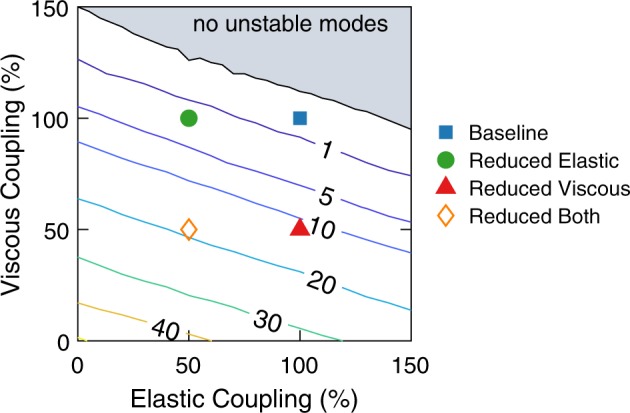
Contour plot of the average number of unstable modes as a function of the value of elastic and viscous coupling parameters as a percent change from the baseline values. *N* = 20 random seeds were analyzed. The symbols identify the parameter sets used in other figures for the baseline, “Reduced elastic”, “Reduced viscous” and “Reduced both” cases.

While Fig. 3 focuses on four different sets of values for the TM parameters, the influence of TM longitudinal coupling parameters on linear stability was also analyzed for more systematic variations in the parameters, where both the TM coupling stiffness and viscosity were allowed to vary between 0% and 150% of the baseline values. The results of this parametric study, shown in Fig. 4, demonstrate that reducing elastic and/or viscous coupling consistently increases the number of linearly unstable modes. This is consistent with results shown in Fig. 3 and confirms that the effects of altering viscoelastic coupling on cochlear stability are robust to changes in TM parameter values.

### Influence of TM longitudinal coupling on the frequencies and amplitudes of SOAEs

**Figure 5:**
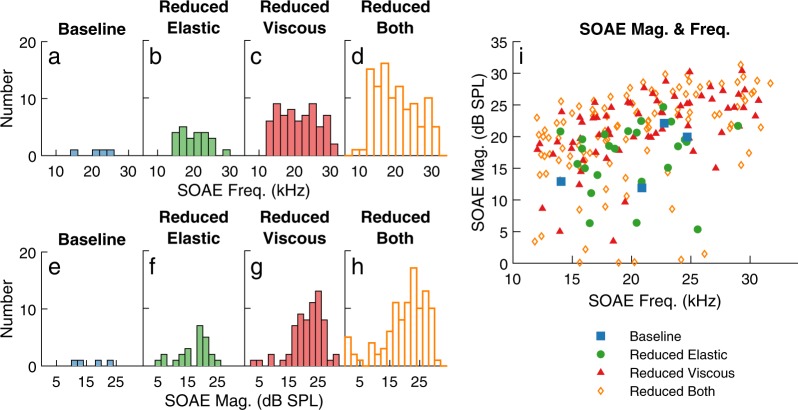
Influence of TM viscoelastic coupling on the amplitude and frequency of SOAEs. (**a**–**d**) Distribution of SOAE frequencies. (**e**–**h**) Distribution of SOAE amplitudes. (**i**) Amplitude vs frequency of SOAEs. The results for the four different models are analyzed for Δ*R* = 0.75% and *N* = 20 different RS.

Analysis of the results obtained for *N* = 20 different RS indicates that for all TM coupling cases, the model predicts that all SOAEs have a frequency between 11 and 32 kHz (Fig. 5a–d). The frequency range of SOAEs predicted by the model corresponds to the frequency range where the cochlear amplifier has the most significant effect on the response of the cochlear model to a pure tone (Supplementary Fig. S2). While reducing TM viscous coupling does not significantly affect the frequency of individual SOAEs (see the discussion of the results of Fig. 2), it broadens the frequency range of SOAEs due to the emergence of additional SOAEs: the frequency range changes from 14 to 26 kHz in the baseline case to 8 to 32 kHz in the “Reduced both” model. While all SOAEs have amplitudes below 26 dB SPL in the “Baseline” and “Reduced elastic” models, the maximum SOAE amplitude is slightly higher in the models with reduced viscous coupling and can reach up to about 31 dB SPL.

### Effect of reducing TM viscoelastic coupling on the cochlear response to low intensity tones

**Figure 6:**
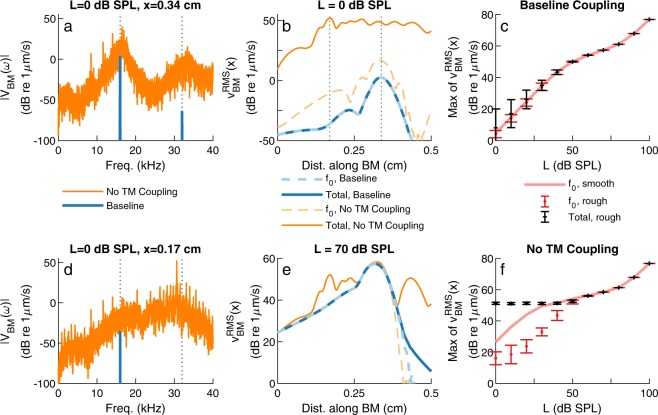
Effect of eliminating TM viscoelastic longitudinal coupling on the BM response to pure tones of stimulus frequency 16 kHz and different sound pressure levels, *L*. In all panels, “*f*_0_” refers to the root mean square (RMS) value of the stimulus frequency component of the BM velocity, ${{v}_{BM}^{RMS}|}_{{f}_{0}}(x)$, while “Total” refers to the RMS value of the total BM velocity, ${{v}_{BM}^{RMS}|}_{tot}(x)$ (see Eq. 3). Two coupling cases are considered: the baseline model and the “No TM coupling” model (${K}_{tm}^{LC}={C}_{tm}^{LC}=0$). (**a**,**d**) Frequency spectrum of the BM at the 16 kHz peak position (*x* = 0.34 cm) and at a more basal position (*x* = 0.17 cm) for RS = 0. The vertical dotted lines indicate the stimulus frequency, *f*_0_ = 16 kHz, and its second harmonic, 2*f*_0_ = 32 kHz. (**b**,**e**) Spatial response of the BM to pure tones of stimulus levels 0 and 70 dB SPL, respectively, for RS = 0. (**c**,**f**) Maximum of the RMS value of the BM velocity for smooth model and rough (Δ*R* = 0.75%) models as a function of sound pressure level. The rough model results in Panels (c,f) include the average of *N* = 10 RS; the error bars correspond to ±1 standard deviation.

The influence of TM viscoelastic longitudinal coupling on the response of the BM to a pure tone of frequency *f*_0_ = 16 kHz was analyzed for two different TM models: (1) the baseline model and (2) a model with no TM longitudinal coupling (“No TM coupling” model), obtained by setting ${K}_{tm}^{LC}=0$ and ${C}_{tm}^{LC}=0$. The Fast Fourier Transform (FFT), *v*_*BM*_(*x, f*), of the time-domain response to a pure tone, *v*_*BM*_(*x, t*), was calculated using MATLAB (The MathWorks, Natick, MA). For the baseline model that does not exhibit any SOAEs, the BM at the 16 kHz peak position primarily vibrates at the frequency of the stimulus, with a secondary peak of lower amplitude at the frequency 2*f*_0_ due to harmonic distortion (Fig. 6a). At a more basal location (*x* = 0.17 cm, Fig. 6d), tuned to 30.5 kHz, the BM also vibrates primarily at 16 kHz but with a much lower amplitude (refer to vertical dotted lines in Fig. 6b). However, the “No TM coupling” model exhibits many linearly unstable modes (more than 50 unstable modes), such that many spectral peaks are observed in the response to a pure tone of low level (Fig. 6a,d). The peak at the frequency of the stimulus is significantly lower than the peaks due to linear instabilities. At each of the two locations, the BM primarily vibrates at frequencies around the corresponding CF, such that, even though the model is excited by a pure tone of frequency 16 kHz, the basal location vibrates at frequencies around 30.5 kHz (Fig. 6d). Because the response of the model remains dominated by instabilities rather than by the response directly evoked by the stimulus, the stimulus would likely be undetectable at low SPL.

To systematically evaluate whether the stimulus frequency component of the response is dominated by vibration at SOAE frequencies, we use the FFT of the BM velocity in response to a pure tone, *V*_*BM*_(*x*, *f*), to calculate the root mean square (RMS) values of the total BM response, ${{v}_{BM}^{RMS}|}_{tot}(x)$, and the RMS value of the stimulus frequency component of the BM velocity response, ${{v}_{BM}^{RMS}|}_{{f}_{0}}(x)$, using the following equations:

3 $${{v}_{BM}^{RMS}|}_{{tot}}(x)=\sqrt{\frac{1}{2}\sum _{f=0}^{{f}_{max}}\,|{V}_{BM}(x,f){|}^{2}}\,{\rm{and}}\,{{v}_{BM}^{RMS}|}_{{f}_{0}}(x)=\frac{|{V}_{BM}(x,{f}_{0})|}{\sqrt{2}}$$

where *V*_*BM*_(*x*, *f*_0_) corresponds to the component of frequency *f*_0_ in the FFT of the time-domain response to a pure tone.

The values of ${{v}_{BM}^{RMS}|}_{tot}$ and ${{v}_{BM}^{RMS}|}_{{f}_{0}}$ are plotted as a function of position in response to a 16 kHz pure tone in Fig. 6b,e. When the model has no unstable modes or SOAEs and thus only vibrates at the stimulus frequency, such as the baseline model shown in Fig. 6b,e, ${{v}_{BM}^{RMS}|}_{tot}$ and ${{v}_{BM}^{RMS}|}_{{f}_{0}}$ nearly exactly match each other since the BM primarily vibrates at the stimulus frequency. At 0 dB SPL (Fig. 6b), eliminating TM longitudinal coupling increases the total RMS value by about 30 dB and the RMS value of the stimulus frequency component of the response, ${{v}_{BM}^{RMS}|}_{{f}_{0}}$, by only about 15 dB (close to its peak). In the case of a 70 dB stimulus (Fig. 6e), the 16 kHz and total RMS velocities of the “No TM coupling” model peak at about the same location and have similar magnitudes, suggesting that the 16 kHz stimulus tone would be detectable.

The maxima of the RMS values of the stimulus frequency of the BM response and of total BM responses are plotted as a function of stimulus level in Fig. 6c,f. If cochlear roughness is ignored (smooth model), both the baseline and “No TM coupling” models exhibit the compressive nonlinearity typically observed in measurements in live animals^24^. The low amplitude response of the smooth “No TM coupling” model is 15–20 dB higher than the smooth baseline model. This increase in the maximum response of the smooth “No TM coupling” model, together with the 15 dB increase in the 16 kHz response, ${{v}_{BM}^{RMS}|}_{{f}_{0}}$, in Fig. 6b indicate an increase in cochlear amplification when TM longitudinal coupling is eliminated. When cochlear roughness is introduced, the maxima of the stimulus frequency and total responses of the baseline model (Fig. 6c) closely follow the smooth model. For the “No TM coupling”, rough models (Fig. 6f), ${{v}_{BM}^{RMS}|}_{{f}_{0}}$ follows a similar trend to the corresponding smooth model, but the magnitude of the maxima are reduced by 10–20 dB at low SPL. The total responses, ${{v}_{BM}^{RMS}|}_{tot}$, exhibit a plateau of nearly constant value below ≈5  dB SPL due to the presence of numerous spontaneous oscillations on the BM. The reduction of the 16 kHz response for the rough model relative to the smooth model may be due to suppression of the stimulus frequency response by these spontaneous oscillations. Additionally, the spontaneous oscillations are so large that the 16 kHz stimulus would be detectable only above ≈5  dB SPL, when the total RMS response begins to be dominated by the stimulus frequency component of the response.

## Discussion

There are several important structures within the cochlea that provide longitudinal coupling: the BM^51^, the TM, and Y-shaped elements formed by the basally slanted OHC, the apically slanted phalangeal process, and the supporting Deiters’ cell^52–54^. In addition to longitudinal coupling of the TM, the model used for this study also includes longitudinal coupling of the BM, which is modeled using an orthotropic plate model (see ref.^18^ and Supplementary Table S1 for the parameter values), but does not take into account the Y-shaped elements. BM longitudinal coupling likely has a relatively limited influence on SOAE generation since it has a relatively weak influence on the response to a pure tone^18^. However, longitudinal coupling by the Y-shaped elements, which play an essential role in cochlear amplification in some models^52–54^, might have a more significant influence on SOAE generation. Because some genetic mutations are known to affect TM longitudinal coupling^41–43,55^, longitudinal coupling of the TM is particularly interesting and was the focus of this work.

Previous work has characterized the material properties of isolated segments of murine TMs based on a transversely isotropic and viscoelastic model within the auditory frequency range^55^. In that study, Lemons *et al*.^55^ reported that the TM stiffness is about 25 times higher in the radial direction than in the longitudinal direction. In the current model, the TM is assumed to vibrate rigidly within each cross-section, which is consistent with the high stiffness in the radial direction. In the model, the viscoelastic coupling between longitudinal cross-section is directly proportional to the shear modulus, *G*_*tm*_, and shear viscosity, *η*_*tm*_, of the TM (Eqs 10 and 11). *G*_*tm*_ and *η*_*tm*_ are material properties that have been directly reported by Lemons *et al*.^55^ and other previous studies^17,42,56^ for the TM of the mouse cochlea. These parameters have values (*G*_*tm*_ ≈ 5 kPa at the 20 kHz location and *η*_*tm*_ = 0.03 Pa · s) that are lower than reported by Lemons *et al*. (*G*_*tm*_ ≈ 70 kPa and *η*_*tm*_ ≈ 0.6 Pa · s), Ghaffari *et al*.^17^ (*G*_*tm*_ ≈ 47 kPa and *η*_*tm*_ ≈ 0.19 Pa · s) and Jones *et al*.^56^ (*G*_*tm*_ ≈ 80 kPa and *η*_*tm*_ ≈ 0.06, *η*_*tm*_ ≈ 0.06 Pa · s) for basal TM segments of the mouse cochlea. While some of these differences could be explained by the difference in the species, it is likely that we underestimate the effects of reducing viscoelastic coupling on cochlear stability. Development of a cochlear model of the mouse cochlea with a more detailed representation of the TM directly based on the reported anisotropic properties of the TM would be a useful extension of the presented research that would potentially allow for more quantitative predictions.

Even though the influence of TM mutations on SOAE generation has only been reported for the mouse cochlea, the model used for this paper corresponds to a different species of rodents, the gerbil, which is a limitation of this study. The occurrence of SOAEs tends to vary among rodents: for example, they are much more common in guinea pigs^57^ than in WT mice^36^. Only one (unsuccessful) attempt to observe SOAEs in the gerbil cochlea has been reported^58^. Reports of successful and unsuccessful attempts to measure SOAEs in large populations of gerbils and other rodents would be useful for validation of theoretical studies. Our baseline model of the gerbil cochlea is not inconsistent with an absence or near absence of SOAEs in the gerbil, since we predict an average of only 0.1 SOAE per ear in the baseline model with Δ*R* = 0.75%: only one cochlear model out of 10 emits 1 SOAE. Prediction of a higher number of SOAEs is only obtained when either (1) Δ*R* is increased to larger values or (2) TM viscoelastic coupling is reduced significantly.

For the model of the gerbil cochlea used in this study, we found very robust conclusions regarding the influence of the shear viscosity and modulus of the TM. When the shear viscosity and/or modulus of the TM is reduced, the numbers of linearly unstable modes and SOAE peaks increase significantly. As seen in Fig. 5, lowering TM viscous or elastic coupling has limited influence on the frequencies of individual SOAEs and causes moderate increases in the amplitude of SOAEs. The increase in the number of SOAEs with reduced viscoelastic coupling is correlated with a sharpening of cochlear tuning^18^ caused by the weakened stabilization by neighboring longitudinal cross-sections. The enhancement of SOAEs when TM viscous coupling is lowered might be due to a decrease in energy dissipation within the TM.

The prediction of enhanced SOAE generation when TM elastic coupling is reduced is in contrast with a previous theoretical study that has investigated the effect of elastic coupling on the spontaneous oscillations of non-mammalian HB^59^. In that work, the authors found that elastic coupling tends to increase the amplitude and sharpen the frequency tuning of spontaneous HB oscillations in the presence of noise. A key difference in our work is that, because our focus is on the mammalian cochlea, we take into account organ-level fluid mechanics, organ of Corti mechanics, and the cochlear traveling wave, while Dierkes *et al*.^59^ only modeled individual nonlinear oscillators coupled by elastic springs. Indeed, SOAE generation is a global phenomenon that requires the collective action of OHCs over a finite extent of the cochlear partition in our model; individual sections of the organ of Corti model are stable (see Supplementary Fig. S3). Additionally, SOAE generation is significantly inhibited if reflection of reverse traveling waves at the stapes is weak (see Supplementary Fig. S7).

Our conclusions regarding the enhancement of SOAEs when TM viscoelasticity is reduced are remarkably similar to observations of increased occurrence of SOAEs in genetically modified mice^36,38^: for example, while SOAEs are uncommon in WT mice (only ∼8.2% emit SOAEs), about 52% of *Tecta*^*Y*1870*C*/+^ mice emit SOAEs^38^. In the *Tecta*^*Y*1870*C*/+^ mice, the TM has reduced shear viscosity and reduced shear stiffness^42,43^ due to the increased porosity of the TM and the total loss of the striated sheet matrix. Our results show that models with both reduced coupling viscosity and stiffness emit significantly more SOAEs than baseline models, as observed in the experiments with the *Tecta*^*Y*1870*C*/+^ mice. Similarly, the occurrence of SOAEs is significantly increased in the *Ceacam16* KO mice^36^; while the mechanical properties of the TM of *Ceacam16* KO mice have not been characterized, our theoretical results suggest that the enhancement of SOAEs in *Ceacam16* KO might also be due to a decreased viscosity and/or stiffness of the TM. Previous work has shown that the *Tectb* KO mutation primarily affects the shear viscosity of the TM, which is about 50% lower than its value in WT TMs^42,55^. Our model predicts that if the mutation only affects the TM shear modulus, *Tectb* KO mice should emit slightly more SOAEs than WT mice, which is consistent with measurements by Cheatham *et al*.^38^.

While our model predictions provide compelling evidence that reducing TM viscoelastic coupling enhances the generation of SOAEs, it does not capture all of the experimental observations regarding SOAE generation in TM mutant mice. For example, the model does not give a mechanism for the increased occurrence of low frequency SOAEs in *Tecta*^*Y*1870*C*/+^ mice^38^. The limited validity of the model at the apex of the cochlea, where OHCs are predicted to have a nearly negligible influence on the BM response to sound (see Supplementary Fig. S2b), is a likely cause for the absence of low frequency SOAEs predicted by the model when TM viscoelastic coupling is reduced. Furthermore, other potential enhancement mechanisms for SOAE generation have been proposed. For example, the emergence of holes in the TM of *Ceacam16* KO mice^36^ and *Tecta*^*Y*1870*C*/+^ mice^44^ could cause the reflection of traveling waves and enhance the generation of SOAEs according to the theory of coherent reflection, as discussed by Cheatham *et al*.^36^. We considered the presence of these holes by adding roughness in the TM viscoelastic longitudinal coupling parameters; we found that roughness in the TM parameters has very limited effect on SOAE generation (see Supplementary Fig. S8). The loss of the Hensen’s stripe in the apical turn of the *Ceacam16* KO mice^36^ could reduce fluid viscous dissipation in the subtectorial space and thereby slightly enhance SOAE generation (see Supplementary Fig. S9). The thicker TM in the apical turn of *Tectb* KO mice^35^ might also affect SOAE generation due to the increased mass and/or to the altered hydrodynamics in the subtectorial space. The enhancement of SOAEs in the *Otoa* KO mice^37^, in which the TM is detached from the spiral limbus, might be due to the change in the mechanical load applied by the TM on the OHC HBs caused by the detachment of the TM^60^.

Remaining on the stable side of a dynamic bifurcation might be desirable to maximize the sensitivity of the cochlea to low level inputs^3^. However, due to the inherent inhomogeneities present in a biological system such as the cochlea, for example due to variations in the OHC morphology and properties, being close to a dynamic instability without generating many SOAEs is challenging. Any small change in the parameters of the cochlea might cause the system to move to the oscillatory regime. Our numerical results (Fig. 6) demonstrate that the ability to detect low amplitude sound would be compromised if the cochlea had many SOAEs; furthermore, limit cycle oscillations of large amplitude on the BM would potentially be heard by the subject and cause objective tinnitus.

Multiple roles have been proposed for the TM in the literature. The TM is essential for the stimulation of inner hair cells^34^. Furthermore, the TM applies an inertial load on the OHC hair bundles that might play a critical role in activating the cochlear amplifier^28,33^. The role of TM viscoelastic coupling in controlling the tuning of the BM has been demonstrated experimentally and theoretically^18,35,41^. The theoretical results from this paper, and the previous experiments in transgenic mice by Cheatham *et al*.^36–38^, suggest that another important function of the TM is to prevent the emergence of limit cycles oscillations of large amplitudes.

## Methods

In the model used for this study, cochlear nonlinearity is the consequence of the nonlinearity of the hair bundle mechanoelectrical transduction current, *i*_*MET*_. *i*_*MET*_ is given by:

4 $${i}_{{MET}}({u}_{\text{hb}{/}\text{rl}})={I}_{{hb}}^{{\max }}[\frac{1}{1+\exp [\,-\,\frac{{u}_{\text{hb}{/}\text{rl}}-{X}_{0}}{{\rm{\Delta }}X}]}-{P}_{0}^{s}],$$

where *u*_*hb*/*rl*_ is the deflection of the hair bundle relative to the reticular lamina, ${I}_{{hb}}^{{\max }}$ is the saturating mechanoelectrical transduction current, ${P}_{0}^{s}$ is the resting open probability of the channel, and *X*_0_ and Δ*X* are constant displacements. Somatic electromotility is modeled by the following linear equations:

5 $${i}_{{ohc}}(t)={G}_{m}{\rm{\Delta }}{\varphi }_{{ohc}}(t)+{C}_{m}{\rm{\Delta }}{\dot{\varphi }}_{{ohc}}(t)-{\varepsilon }_{3}{\dot{u}}_{{ohc}}^{{comp}}(t),$$

6 $${F}_{{ohc}}(t)={K}_{{ohc}}{u}_{{ohc}}^{{comp}}(t)+{\varepsilon }_{3}{\rm{\Delta }}{\varphi }_{{ohc}}(t),$$

where *i*_*ohc*_, Δ*ϕ*_*ohc*_ and *F*_*ohc*_ are the perturbations in the somatic current, transmembrane potential and electromechanical force from their resting values, respectively; ${u}_{{ohc}}^{{comp}}$ is the OHC compression; *G*_*m*_ and *C*_*m*_ are the basolateral conductance and capacitance, respectively; *ε*_3_ is the electromechanical coupling coefficient; *K*_*ohc*_ is the OHC stiffness. OHC electromotility is the only active mechanism included in the model; HB motility is not taken into account.

As shown in^45^, the Kirchoff equation for the electrical model of the OHC shown in Fig. 1 couples the OHC transmembrane potential, Δ*ϕ*_*ohc*_, to the transduction current, *i*_*MET*_(*t*):

7 $${C}_{a}{\rm{\Delta }}{\dot{\varphi }}_{hb}(t)+\frac{1}{{R}_{a}^{0}}{\rm{\Delta }}{\varphi }_{hb}(t)+{i}_{{MET}}(t)-{i}_{{ohc}}(t)=0$$

8 $${C}_{a}{\rm{\Delta }}{\dot{\varphi }}_{hb}(t)+(\frac{1}{{R}_{a}^{0}}+\frac{1}{{R}_{eq}}){\rm{\Delta }}{\varphi }_{hb}(t)+\frac{1}{{R}_{eq}}{\rm{\Delta }}{\varphi }_{ohc}(t)={i}_{{MET}}(t)$$

where ${R}_{a}^{0}$ and *C*_*a*_ are the apical resistance and capacitance, respectively, Δ*ϕ*_*hb*_ is the perturbation in the hair bundle potential from its resting value, and *R*_*eq*_ is given by

9 $${R}_{eq}={R}_{tl}+{R}_{vl}+{R}_{vm},$$

where *R*_*tl*_, *R*_*vl*_, and *R*_*vm*_ are the resistances of the current flowing from the scala tympani to ground, from the scala vestibuli to ground, and from the scala vestibuli to ground, respectively^61^.

The TM has two DOFs per cross-section (shear and bending modes); the TM cross-section is assumed to vibrate as a rigid body. The governing equation for mode *tmj* (where *j* = *s* and *b* to denote the shear and bending modes, respectively) is of the following form:

10 $${F}_{\text{hb}{/}\text{tmj}}(x)={K}_{{tmj}}{u}_{{tmj}}+{C}_{{tmj}}{\dot{u}}_{{tmj}}+{M}_{{tmj}}{\ddot{u}}_{{tmj}}-\frac{\partial }{\partial x}({K}_{tm}^{LC}\frac{\partial {u}_{{tmj}}}{\partial x}+{C}_{tm}^{LC}\frac{\partial {\dot{u}}_{{tmj}}}{\partial x})+{C}_{f}{\delta }_{j}{\dot{u}}_{hb}$$

where *x* is the distance from the stapes along the longitudinal axis, *F*_hb/tmj_(*x*) is the force per unit length applied by the OHC HBs on mode *tmj*, *M*_*tmj*_ is the mass per unit length of mode *tmj*. The TM model includes locally reacting parameters (the stiffness, *K*_*tmj*_, and damping coefficient, *C*_*tmj*_, of the attachment of the TM to the spiral limbus) and longitudinal coupling parameters (the TM coupling stiffness, ${K}_{tm}^{LC}$, and viscosity, ${C}_{tm}^{LC}$). ${K}_{tm}^{LC}$ and ${C}_{tm}^{LC}$ are proportional to shear modulus, *G*_*tm*_, and shear viscosity, *η*_*tm*_, of the TM:

11 $${K}_{tm}^{L}C={G}_{tm}{A}_{tm}\,{\rm{and}}\,{C}_{tm}^{L}C={\eta }_{tm}{A}_{tm}$$

where *A*_*tm*_ is the TM cross-section area. Due to the importance of TM longitudinal coupling for this work, longitudinal coupling was added to the TM bending mode (using the same coupling stiffness and viscosity as the TM shear mode). Damping from the fluid in the subtectorial space is taken into account by the presence of a shear force proportional to the hair bundle velocity, ${\dot{u}}_{hb}$, with an effective damping coefficient of value *C*_*f*_, in Eq. 10 for the TM shear mode (δ_*j*_ = 1 if *j* = *s* and δ_*j*_ = 0 if *j* = *b*). The parameter values, given in the SI Appendix, were tuned in order to accurately represent cochlear physiology in a commonly studied mammalian species, the gerbil (see Supplementary Figs S1 and S2).

Cochlear roughness is included by adding random variations of small amplitudes to the baseline value of the OHC electromechanical coupling coefficient, *ε*_3_(*x*), (as we previously introduced to model the response of the cochlea to an acoustic click and fine structure in distortion product otoacoustic emissions)^45,47^:

12 $${\varepsilon }_{3}(x)={\varepsilon }_{3}(x){|}_{{smooth}}\times [1+{\rm{\Delta }}R\times r(x)]$$

where ${\varepsilon }_{3}(x){|}_{{smooth}}$ is the value of *ε*_3_(*x*) in the smooth model, Δ*R* is a constant number that scales the amplitudes of the deviation from the smooth trend, and *r*(*x*) is a number generated by a random number generator based on a normal distribution of average value 0 and standard deviation 1.

As described in^45^, the model equations are written in a state-space formulation of the following form:

13 $$\dot{{\rm{v}}}={\rm{Av}}+\text{nl}(v)+{\rm{B}}{F}_{s}(t)$$

where *v* is the state-space vector, *A* and *B* are matrices, *nl* is a vector that depends on *v*, and *F*_*s*_ is the applied force on the stapes. The poles of the system, *λ*, correspond to the eigenvalues of *A*. The response to an acoustic click was obtained by solving Eq. 13 using MATLAB (The MathWorks, Natick, MA) function ode45. The model was first simulated for a long duration (100 ms to 2 s) so that the steady-state response is obtained. The spectrum of the ear canal pressure was computed by taking the Fast Fourier Transform over 500 ms of the pressure in the scala vestibuli near the stapes and by substracting 35 dB to account for the reverse transmission of sound through the gerbil middle ear^62^.

## Supplementary information

Supplementary Information

## Data Availability

Simulation data generated for this study are available from the corresponding author.
