# Supplementary material for: Reducing tectorial membrane viscoelasticity enhances spontaneous otoacoustic emissions and compromises the detection of low level sound
Source: Sci Rep. 2019 May 16;9:7494. doi: 10.1038/s41598-019-43970-5 (PMC6522542; doi:10.1038/s41598-019-43970-5)
Supplement: Supplementary file 1 — Supplementary Information [file 41598_2019_43970_MOESM1_ESM.pdf]

Supporting Information
Reducing tectorial membrane viscoelasticity enhances spontaneous otoacoustic emissions and compromises the detection of low level sound

Thomas Bowling[1], Charlsie Lemons[1], and Julien Meaud[1,2,*]

[1] *G.W.W. School of Mechanical Engineering, Georgia Institute of Technology, 771 Ferst Drive, Atlanta, Georgia 30332, USA.*
[2] *Petit Institute for Bioengineering and Bioscience, Georgia Institute of Technology, Atlanta, GA 30332, USA.*
[*] *julien.meaud@me.gatech.edu*

April 25, 2019

1 Supporting materials and methods

1.1 Cochlear model Parameters

The addition of longitudinal coupling to the TM bending mode required a slight modification to the model parameters compared to our previous work [1]. The numerical values of the present cochlear model parameters are given in Tables S1 and S2. Some of these parameters appear in Eqs. 4-13 of the manuscript. The other equations of the model are given in previous papers [22, 14, 17, 1].

1.1.1 Mechanical Parameters

Table S1: Mechanical parameters for the cochlear model (where x is the longitudinal position in cm). Parameters denoted * are defined per unit length.

Param.	Description	Value	Ref.
K_{bm}	BM stiffness *	$18.4\exp(-7.54x)\cdot 10^5$ N/m^2	assumed
D_{xx}	BM plate bending stiffness (xx) *	$10^{-10}\exp(-0.5x)$ N.m	[14]
D_{xy}	BM plate bending stiffness (xy) *	$10^{-10}\exp(-0.5x)$ N.m	[14]
D_{shear}	BM plate bending stiffness (shear) *	$4.3\exp(-0.5x)\cdot 10^{-11}$ N.m	[14]
K_{tms}	TM shear stiffness *	$2.31\exp(-1.32x^2-6.42x)\cdot 10^5$ N/m^2	assumed
K_{tmb}	TM bending stiffness *	$3.84\exp(-7.54x)\cdot 10^4$ N/m^2	assumed
K_{rl}	RL stiffness *	$2.78\exp(-7.54x)\cdot 10^3$ N/m^2	assumed
K_{ohc}	OHC stiffness *	$5.07\exp(-7.54x)\cdot 10^3$ N/m^2	assumed
K_{hb}	HB stiffness *	$291\exp(-7.54x)$ mN/m	assumed
M_{bm}	BM mass *	$2.8\cdot 10^{-7}$ kg/m	[5]
M_{tms}	TM shear mass *	$3.58\exp(1.58x)\cdot 10^{-6}$ kg/m	based on [24, 25]
M_{tmb}	TM bending mass *	$1.46\exp(2.15x)\cdot 10^{-6}$ kg/m	based on [24, 25]
c_{bm}	BM damping coefficient *	$8.5\cdot 10^{-2}$ N.s/m^2	assumed
c_{hb}	HB damping coefficient	$\eta_f \frac{L_{tm}}{3L_{hb}}$, where $\eta_f = 1.0\cdot 10^{-3}$ N.s/m^2 is the viscosity of the fluid	[14]
c_{tmb}	TM bending damping coefficient *	0.1 N.s/m^2	assumed
c_{tms}	TM shearing damping coefficient *	$3\cdot 10^{-3}$ N.s/m^2	assumed
G_{tm}	TM modulus	$7.0\exp(-3.75x)$ kPa	[25, 14]
η_{tm}	TM viscosity	0.03 Pa.s	based on [6]
ρ_f	fluid density	1000 kg/m^3	

1.1.2 Electrical Parameters and Model Formulation

The saturating mechanoelectrical transduction current, I_{hb}^{max}, appears in Eq. 4 of the manuscript. I_{hb}^{max}, is related to the parameters given in Table S2 by the following equation:

$$I_{hb}^{max} = G_{hb}^{max} \Delta V_{hb}^{0} \tag{S1}$$

where G_{hb}^{max} is the saturating hair bundle mechanoelectrical conductance and ΔV_{hb}^{0} is the resting value of the difference between the scala media potential and intracellular OHC potential.

As discussed in [15], cochlear amplification depends on the slope of the mechanoelectrical conductance vs HB deflection function; as in our previous work [1], the parameters of the mechanoelectrical transduction channel were chosen such that the sensitivity of the BM response is similar to experimental measurements. Because experimental measurements have shown that the mutations have very limited effect on the cochlear microphonic in $Tecta^{Y1870C/+}$ and $Tectb$ KO mice [11, 26] and on cubic and quadratic distortion product otoacoustic emissions in the *Ceacam16* KO mice [2], the operating point of the transduction channel was assumed to be the same for all models.

Table S2: Electrical parameters for the model (x in cm).

Parameters	Description	Value	Ref.
C_m	Basolateral capacitance	$52.35x$ pF	based on [10]
G_m	Basolateral conductance	$64 - 49.6x$ nS	based on [10]
ϵ_3	Electromechanical coupling coefficient	$1.04 + 0.36x$ N/m/mV	based on [9]
$1/R_a^0$	apical resistance	$172\exp(-2.05x)$ nS	[16]
G_a^{max}	saturating HB conductance	Interpolated from<br>479 nS at x=0cm<br>438 nS at x=0.13cm<br>422 nS at x=0.15cm<br>331 nS at x=0.224 cm<br>148 nS at x=0.44 cm<br>60 nS at x=0.67 cm<br>14.8 nS at x=1.12 cm	assumed
C_a	apical capacitance	50 nF/m	based on [4]
R_{tl}	resistance from ST to ground	4 Ωm	based on [29]
R_{vm}	resistance from SV to SM	25 Ωm	based on [29]
R_{vl}	resistance from SV to ground	10 Ωm	based on [29]
P_0^s	resting probability	0.4	[16]
ΔV_{hb}^0	resting value of potential difference between scala media and intracellular OHC potential	$150 - 10x$ mV	[16]

1.2 Middle ear model and parameters

As in our previous work [1], the cochlear model is coupled to a one DOF model of the middle ear with mass, damping and stiffness coefficients. While sound transmission through the middle ear can be represented by more realistic multi-DOF models [19, 12], the advantage of a simpler model is that it makes it possible to easily vary the reflection of reverse waves at the stapes to study the influence of the middle ear properties on SOAE generation (see for example Fig. S7). In the frequency domain, the reverse middle ear impedance (*i.e*, the impedance looking out from the stapes in the reverse direction [21]) is given by:

$$Z_{meR} = \frac{1}{A_s^2}\left[M_s i\omega + C_s + \frac{K_s}{i\omega}\right] \tag{S2}$$

where M_s, C_s, and K_s are the mass, damping coefficient, and stiffness coefficient of the stapes, respectively; ω is the radian frequency and A_s is the area of the stapes footplate.

In the theory of coherent reflection [27], the reflection of the reverse traveling wave by the stapes plays a critical role in SOAE generation: SOAEs are expected to be generated only when the amplitude of the stapes reflection coefficient is sufficiently close to 1. This hypothesis was tested in our model. The stapes reflection coefficient, R_{st}, can be calculated using the equation [28, 1]:

$$R_{st} = \frac{Z_{meR}/Z_c^* - 1}{Z_{meR}/Z_c + 1} \tag{S3}$$

where Z_c is the input impedance of the cochlear model, and * denotes the complex conjugate. Two parameter sets for the middle ear model, given in Table S3, were considered: a baseline parameter set (used in all of the numerical results except for some results in Fig. S7), in which $|R_{st}|$ is approximately equal to 0.7 from 10 to 25 kHz; a low $|R_{st}|$ parameter set (used only for Fig. S7) in which $|R_{st}|$ is below 0.2 from 10 to 25 kHz (see Fig. S2 in [1]).

Table S3: Mechanical parameters for the middle ear model.

Parameters	Description	Baseline	Low $\|R_{st}\|$
M_s	Stapes mass (kg)	3.0×10^{-7}	6.6×10^{-7}
C_s	Stapes damping coefficient (N.s/m)	6.0×10^{-2}	3.4×10^{-1}
K_s	Stapes stiffness (N/m)	5.0×10^{2}	2.3×10^{4}

1.3 Calibration of the model: comparison of pure tone response to experimental data

At a longitudinal position, x, and frequency, ω, the gain of the BM velocity relative to the stapes velocity is defined as

$$G_{bm}(x,\omega) = \frac{v_{bm}(x,\omega)}{v_s(\omega)}, \tag{S4}$$

where v_{bm} and v_s are the velocities of the BM and stapes, respectively. The pure tone response of the smooth model is compared to experimental data from Refs. [20] and [23] in Fig. S1. As mentioned earlier, model parameters were adjusted so that the gain of

the model matched that reported from experimental measurements. At the 34.6 kHz peak position (CF=34.6 kHz, Fig. S1a), the model results are similar to the experimental data in terms of the sharpness of the peaks and relative levels of the gain (both show a $\approx$ 21 dB difference between low and high SPL). At CF=13.3 kHz (Fig. S1b), the both model and experiment show a $\approx$27 dB difference between low and high SPL and the peaks are similarly broad. However, there is a noticeable difference of 11 dB between the model and experiment that is possibly due to a mismatch in the structural properties or assumptions of the model.

As is the case with many cochlear models (e.g. [30, 1, 18]), the phase rolls-off or decreases somewhat more rapidly than what is observed experimentally (Figs. S1c and S1d) despite effort during calibration to mitigate this. The mismatch between the model and the experimental data is, however, noticeably smaller than that of the previous version of the model (Fig. 3 in [1]). The pure tone response of the smooth model at all longitudinal positions with the place-frequency map, ratio of the BM gain at CF, and quality factor, Q_{10dB} are shown in Fig. S2. The model results in Fig. S2 were obtained using a linear formulation of the model; the active and passive models represent the model responses at low and high stimulus levels, respectively. The passive model is obtained by setting $G_{hb}^{max} = 0$ in Eq. S1. As shown in the figures, the model predictions agree well with the measurements from multiple longitudinal locations.

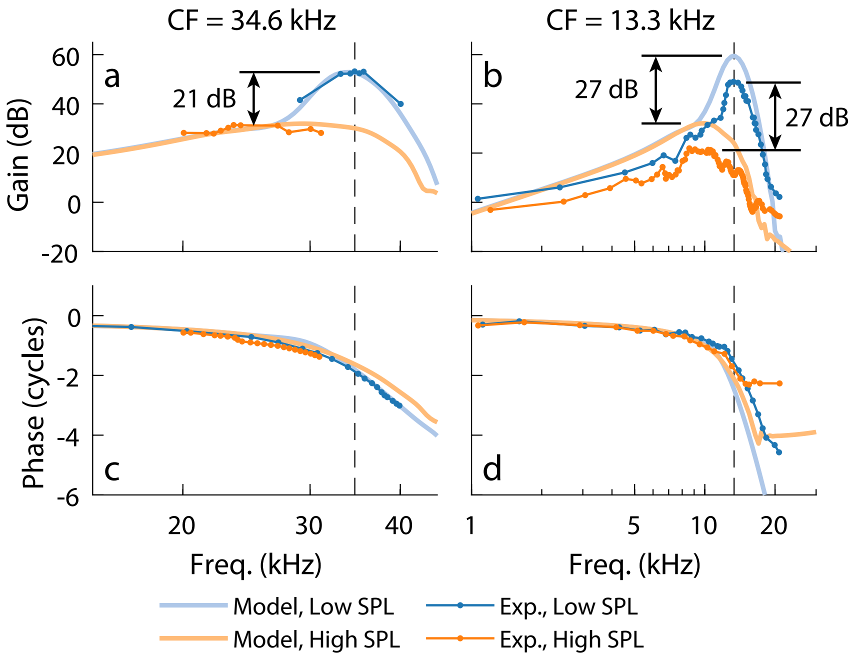

Figure S1: Comparison of BM pure tone response for the current model and experimental data taken from Refs. [23] and [20]. (**a**) and (**b**) Gain of the BM velocity relative to the stapes velocity. (**c**) and (**d**) Phase of the BM velocity relative to the stapes velocity. (**a**) and (**c**) Experimental data from Ref. [20] is given. (**b**) and (**d**) Experimental data from Ref. [23] is given. (**a**) and (**c**) Results for 30- and 90 dB SPL and 50- and 90 dB SPL stimuli, respectively. (**b**) and (**d**) Results for 30 and 100 dB SPL stimuli. The vertical dashed lines denote CF.

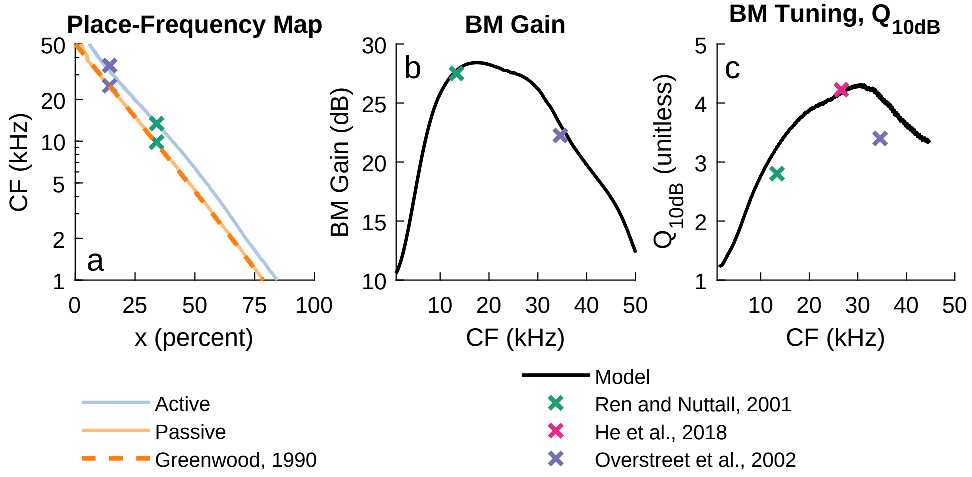

Figure S2: Comparison of BM pure tone response for model and experimental data from [7, 23, 20, 8]. (**a**) Place-frequency map of the passive and active models with data from [7, 23, 20]. (**b**) Gain of the active model relative to the passive model. (**c**) Quality factor, Q_{10dB}, of the tuning of the BM. Model results are compared with measurements from the 13 kHz [23], 27 kHz [8], and 34 kHz [20] best places. The stimulus levels used for computing the gain from the experimental data at the 13 and 34 kHz positions were 30 and 100; 30 and 90 dB SPL, respectively. The quality factors for the experiments were computed from the responses to a 30 dB SPL stimulus.

2 Supporting results

2.1 Stability of organ of Corti model

To determine whether the spontaneous oscillations in the model were generated by local instabilities or a global phenomenon, the linear stability of an isolated longitudinal cross-section of the organ of Corti was analyzed (Fig. S3). This organ of Corti model neglects longitudinal coupling and fluid loading. Both the fully active model and a passive model (obtained by setting the mechanoelectrical transduction current to 0) were analyzed. As seen in Fig. S3b, both the passive and active organ of Corti models are linearly stable. The parameters of the cochlear model vary spatially (Tables S1 and S2), so the stability of the organ of Corti at all other positions was analyzed. For all positions along the BM, the organ of Corti model remained stable. This is consistent with the theory that SOAEs are caused by a global phenomenon instead of local instability.

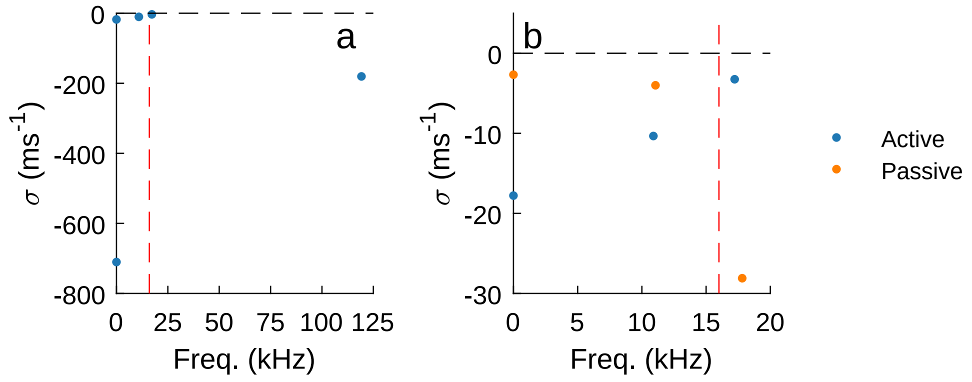

Figure S3: Stability of the organ of Corti model at the 16 kHz best place. CF is denoted by the vertical dashed line. (**a**) Linear stability diagram for the active model. (**b**) Zoom-in on the linear stability for low frequencies and frequencies near CF for the active and passive models.

2.2 Influence of mesh on linear stability

Throughout the results shown in the manuscript, a finite element mesh with elements of longitudinal length $\Delta x = 25$ μm long was used (total of 448 elements along the BM). Furthermore, the roughness function, $r(x)$, is assumed to be a piecewise linear function with breakpoints that are separated by a distance Δx_r. For the results shown in the manuscript, Δx_r was chosen to be also equal to 25 μm.

The influence of Δx and Δx_r on the results was examined. Two different cases were considered: (1) Δx_r was fixed at 25 μm and the element size, Δx, was varied, shown in Fig. S4 and (2) the roughness lengthscale, Δx_r, was varied for a fixed element size ($\Delta x = 25$ μm) shown in Figs. S5.

The results for the first case (Fig. S4), indicate a slight decrease in stability for models with $\Delta x = 12.5$ μm long elements. When the element size is increased to $\Delta x = 50$ μm, there is a noticeable decrease in the number of linearly unstable models (Fig. S4d). The same roughness variations were used to allow for a meaningful comparison (Fig. S4a and S4b); using different roughness variations would result in slightly different numbers of linearly unstable modes. For many of the models considered, using the finer mesh results in at most one additional linearly unstable mode than using the mesh used in the manuscript. Shown in Fig. S4c is a less common case, in which two nearly unstable modes for $\Delta x = 25$ μm (indicated by the arrows) become unstable when the element size is reduced to $\Delta x = 12.5$ μm. Such a small decrease in stability (increase in σ) for the finer mesh also affects n_{inst} when $N = 20$ models are considered, as shown in Fig. S4d. Despite the slight decrease in stability for the finer mesh, the results in Fig. S4d are consistent with results shown in Figs. 2-5 and confirm that the effects of altering viscoelastic coupling on cochlear stability do not change if the size of the element is doubled or divided by two.

Varying the length scale of the roughness, Δx_r, results in different roughness functions, $r(x)$ (Fig. S5). Using a roughness length scale of 10-25 μm is reasonable given the value of the OHCs diameter (8-10 μm [3]) and the variability in OHC organization [13]. While identifying the related individual poles for the three meshes was straightforward in Fig. S4c,

there is no such correlation between poles for the results shown in Fig. S5c since different $r(x)$ are used. This difference is due to the roughness having very different spatial variations (Fig. S5a and S5b). More importantly, the average numbers of linearly unstable modes is not affected much by the changes in Δx_r (Fig. S5d). The results of Fig. S5d show that reducing TM viscoelastic coupling results in a reduction in cochlear stability and an increase in the average number of linearly unstable modes at all Δx_r values that were considered.

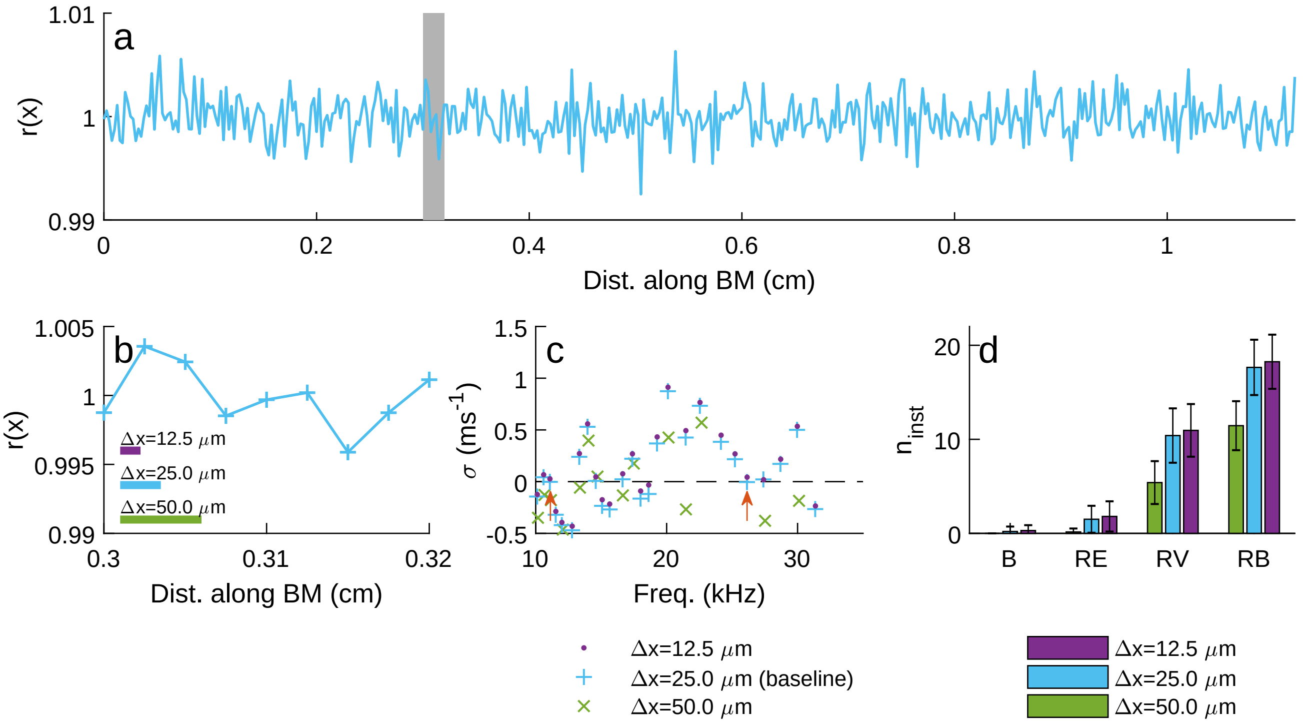

Figure S4: Influence of the element size, Δ_x, of the finite element mesh on linear stability for $\Delta R = 0.75\%$. Effects on linear stability for various meshes of different element size with a fixed roughness length scale ($\Delta x_r = 25\ \mu$m). (**a-b**) Roughness variations, $r(x)$ (Eq. 12), for RS=2. The narrow vertical shaded region in Panel (**a**) corresponds to the region shown in Panel (**b**). (**b**) The scale bars represent the element lengths considered (12.5, 25, and 50 μm). (**c**) Linear stability diagram for the "Reduced Both" model for RS=2. (**d**) Average number of linearly unstable models, n_{inst}, for N=20 RS for the four compling cases, "Baseline" (B), "Reduced Elastic" (RE), "Reduced Viscous" (RV), and "Reduced Both" (RB). The error bar corresponds to $\pm$ one standard deviation.

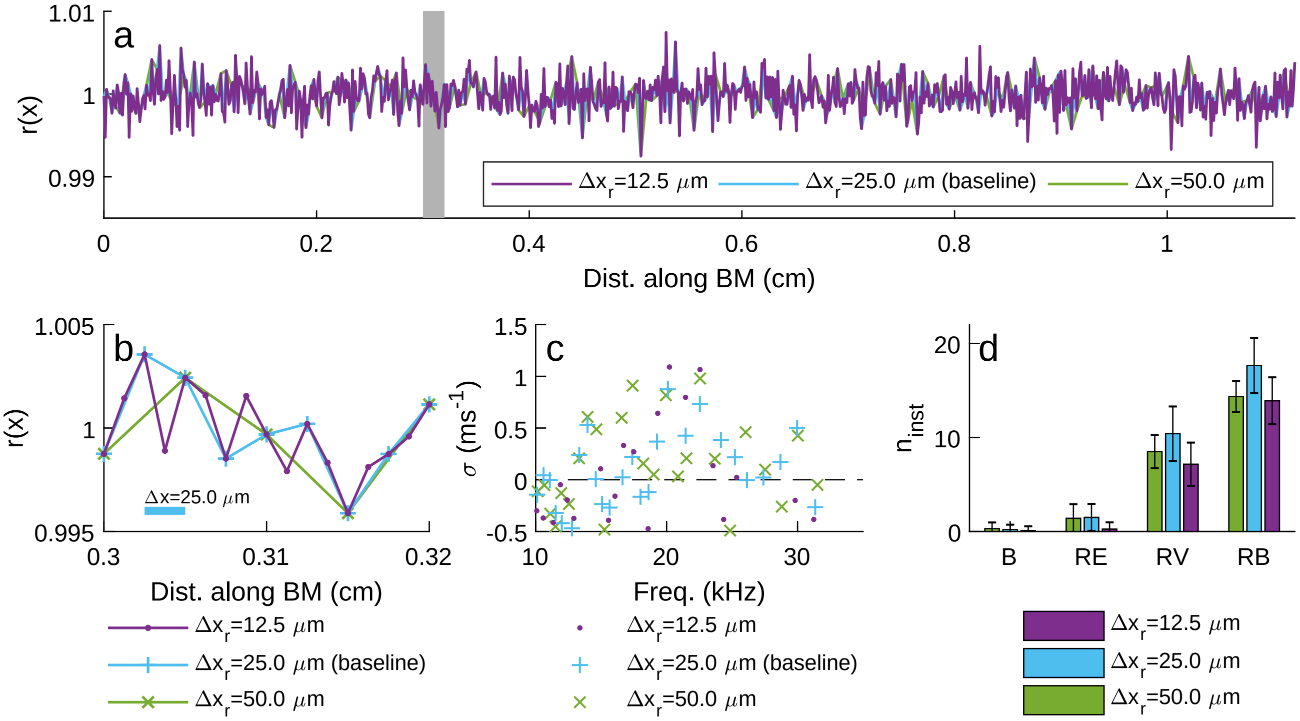

Figure S5: Influence of the roughness length scale (Δx_r) on linear stability for a fixed Δx = 25 μm mesh (indicated by the scale bar in Panel (**b**)). Other details are the same as Fig. S4.

2.3 Influence of large roughness variations on linear stability

In Fig. 3c, the influence of varying the amplitude of the random perturbations, ΔR, on the average number of linearly unstable modes was examined for a relatively narrow range of ΔR values ($\Delta R \leq 1\%$). For larger random perturbations ($1\% \leq \Delta R \leq 40\%$), shown in Fig. S6, the average number of unstable modes continues to increases as ΔR is increased, albeit at a slightly lower rate once ΔR exceeeds 5%. In all cases, for a given random perturbation amplitude, reducing TM viscoelastic coupling results in a reduction in cochlear stability and an increase in the average number of linearly unstable modes.

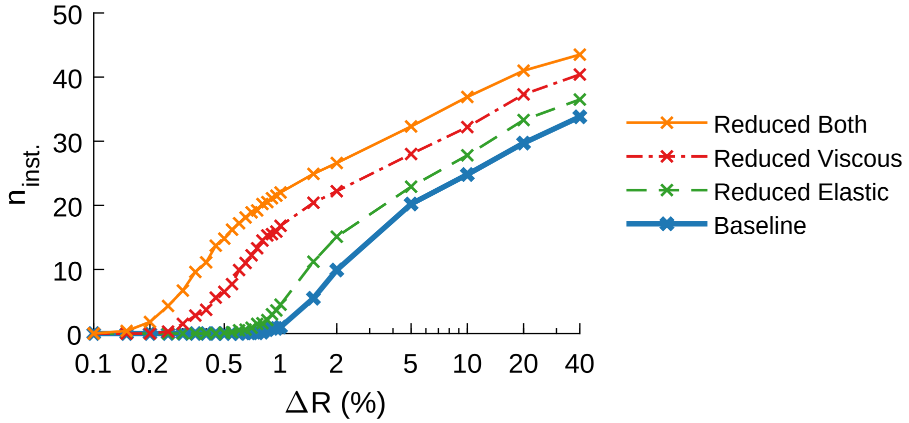

Figure S6: Influence of the amplitude of the random perturbation, ΔR, on the number of linearly unstable modes for $N = 10$ different RS.

2.4 Influence of stapes reflectance on cochlear stability and SOAEs

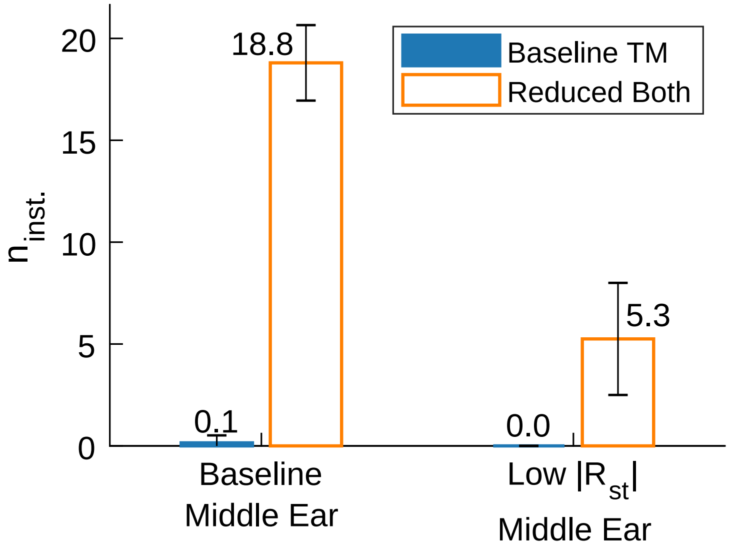

Figure S7: Influence of the stapes reflectance on the average number of linearly unstable modes. $N = 20$ random seeds were analyzed for each case. The error bar corresponds to $\pm$ one standard deviation.

The results for the linear stability of the models, shown in Fig. S7, demonstrate that unstable modes are significantly reduced when the magnitude of the stapes reflection coefficient, $|R_{st}|$, is low. Since SOAEs are only generated when linear unstable modes are present, this implies that SOAE generation requires the stapes reflection coefficient to have a sufficiently high magnitude. This is a consistent with the theory that are SOAEs are a global phenomenon rather than locally generated.

2.5 Influence of roughness in the TM coupling properties on cochlear stability and SOAEs

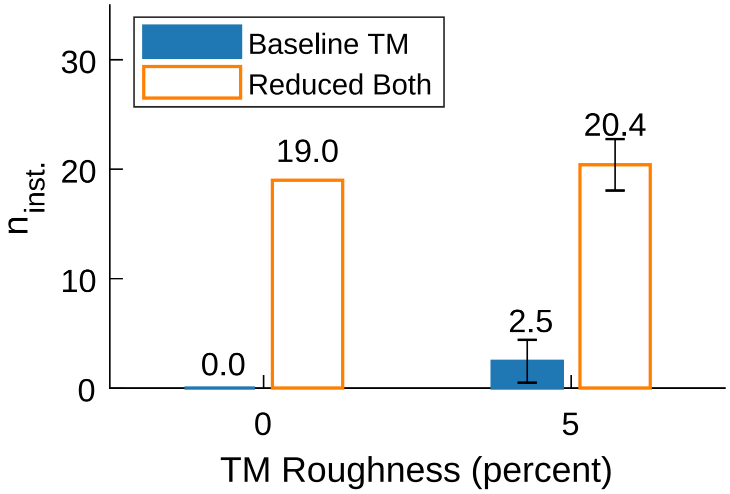

Figure S8: Modeling the effect of holes in the TM on linear stability. The random seed (RS) of the electromechanical coupling coefficient is fixed to RS=13 for these results. $N = 20$ random seeds were used for the roughness in the TM properties. The error bar corresponds to $\pm$ one standard deviation.

Because the presence of holes has been reported in the TM of *Ceacam16* KO and $Tecta^{Y1870C/+}$ mice [2, 11], random inhomogeneities in the TM properties were considered. These inhomogeneities could potentially enhance the generation of reflection-type otoacoustic emissions, including SOAEs. These inhomogeneities were introduced by using the following equations of the longitudinal coupling stiffness and viscosity of the TM:

$$\begin{aligned} K^{LC}_{tm}(x) &= K^{LC}_{tm}(x)|_{smooth} \times \left[1 + \Delta R_{tm} \times (r_{tm}(x) - 1)\right] \\ C^{LC}_{tm}(x) &= C^{LC}_{tm}(x)|_{smooth} \times \left[1 + \Delta R_{tm} \times (r_{tm}(x) - 1)\right]. \end{aligned} \tag{S5}$$

where ΔR_{tm} is the amplitude of the random perturbation (which was set to 5%, which is somewhat similar to the percentage of the TM area occupied by holes in the TM of *Ceacam16* KO mice [2]), $r_{tm}(x)$ is a number generated by a random number generator based on a normal distribution of average value 0 and standard deviation 1. Since roughness due to holes in the TM or to inhomogeneities in the OHC properties arises from different mechanisms, different random seed numbers were used for $r_{tm}(x)$ in Eq. S5 and $r(x)$ in Eq. 12. The results shown in Fig. S8 show that adding roughness to the TM properties has a limited effect on the linear stability of the models: the $N = 20$ different models with roughness in the TM properties with the baseline value of the TM parameters have $n_{inst.} = 2.5$ unstable modes, while the model without roughness in the TM parameters has no unstable modes; for the model with reduced viscosity, the average number of instabilities is slightly higher in the models with roughness in the TM parameters (average=20.4) than in the model without TM roughness (which has 19 unstable modes).

2.6 Influence of other TM parameters on cochlear stability and SOAEs

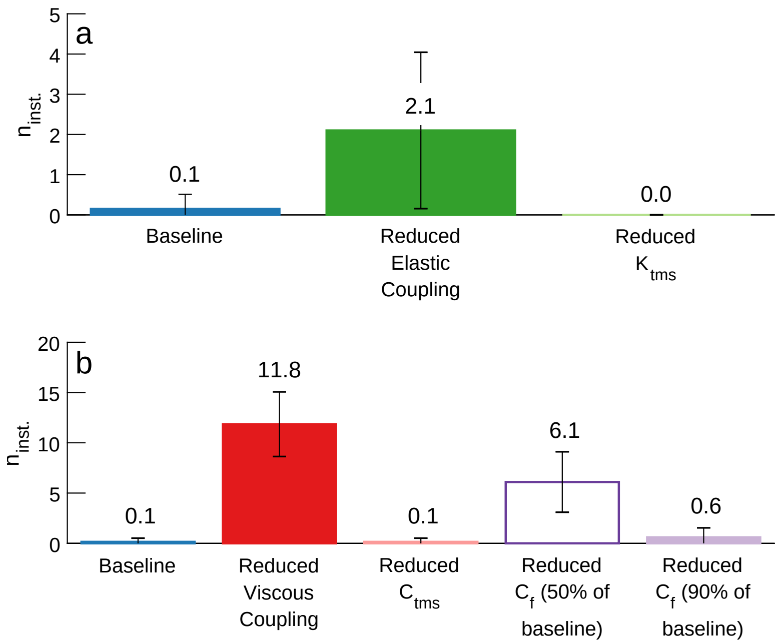

Figure S9: Effect of changes in other parameters on the average number of unstable modes. (**a**) Change in the stiffness parameters. (**b**) Changes in the damping parameters. The error bar corresponds to ± one standard deviation. $N = 20$ different random seeds were analyzed. The change corresponds to 50% of the baseline value for C_{tm}^{LC} and C_{tms}, 50% of the baseline value for K_{tm}^{LC} and K_{tms}; 50% and 90% of the baseline value for C_f.

Because TM mutations might affect other properties of the TM, the influence of other TM model parameters on linear stability was also analyzed (Fig. S9). For example, given the effect of the mutations on the intrinsic properties of the TM, TM mutations might also cause a reduction in the stiffness or viscosity of the attachment of the TM to the spiral limbus. Reducing the stiffness of the attachment to the spiral limbus, K_{tms}, reduces the average number of linearly unstable modes; this effect is opposite to the increase in linearly unstable modes observed when the TM longitudinal coupling elastic stiffness is reduced. Reducing the viscosity of the attachment of the TM to the spiral limbus slightly increases the average number of linearly unstable modes; however, reducing the TM viscosity has a much more significant effect. It is possible that due to the loss of the Hensen's stripe or broadening of the subtectorial space, TM mutations tend to reduce power dissipation in the subtectorial space, which would affect the effective damping coefficient due to fluid viscosity in the subtectorial space, C_f, and would slightly increase the number of instabilities.

References

[1] T. Bowling and J. Meaud. Forward and reverse waves: modeling distortion products in the intracochlear fluid pressure. *Biophys. J.*, 114(3):747–757, Feb. 2018.

[2] M. A. Cheatham, R. J. Goodyear, K. Homma, P. K. Legan, J. Korchagina, S. Naskar, J. H. Siegel, P. Dallos, J. Zheng, and G. P. Richardson. Loss of the tectorial membrane protein CEACAM16 enhances spontaneous, stimulus-frequency, and transiently evoked otoacoustic emissions. *The Journal of Neuroscience*, 34(31):10325–10338, 2014.

[3] P. Dallos. Overview: cochlear neurobiology. In P. Dallos, A. N. Pupper, and R. R. Fay, editors, *The cochlea*, pages 1–43. Springer, 1996.

[4] P. Dallos and B. N. Evans. High-frequency motility of outer hair cells and the cochlear amplifier. *Science*, 267(5206):2006, 1995.

[5] C. Fernández. Dimensions of the cochlea (guinea pig). *The Journal of the Acoustical Society of America*, 24(5):519–523, 1952.

[6] R. Ghaffari, A. J. Aranyosi, and D. M. Freeman. Longitudinally propagating traveling waves of the mammalian tectorial membrane. *Proceedings of the National Academy of Sciences*, 104(42):16510–16515, 2007.

[7] D. D. Greenwood. A cochlear frequency-position function for several species - 29 years later. *The Journal of the Acoustical Society of America*, 87(6):2592–2605, 1990.

[8] W. He, D. Kemp, and T. Ren. Timing of the reticular lamina and basilar membrane vibration in living gerbil cochleae. *eLife*, 7:e37625, 2018.

[9] K. H. Iwasa and M. Adachi. Force generation in the outer hair cell of the cochlea. *Biophysical Journal*, 73(1):546–555, 1997.

[10] S. L. Johnson, M. Beurg, W. Marcotti, and R. Fettiplace. Prestin-driven cochlear amplification is not limited by the outer hair cell membrane time constant. *Neuron*, 70(6):1143–1154, 2011.

[11] P. K. Legan, V. A. Lukashkina, R. J. Goodyear, A. N. Lukashkin, K. Verhoeven, G. Van-Camp, I. J. Russell, and G. P. Richardson. A deafness mutation isolates a second role for the tectorial membrane in hearing. *Nature Neuroscience*, 8(8):1035–1042, August 2005.

[12] C. Lemons and J. Meaud. Middle-ear function in the chinchilla: Circuit models and comparison with other mammalian species. *The Journal of the Acoustical Society of America*, 140(4):2735–2753, 2016.

[13] B. Lonsbury-Martin, G. Martin, R. Probst, and A. Coats. Spontaneous otoacoustic emissions in a nonhuman primate. ii. cochlear anatomy. *Hearing research*, 33(1):69–93, 1988.

[14] J. Meaud and K. Grosh. The effect of tectorial membrane and basilar membrane longitudinal coupling in cochlear mechanics. *The Journal of the Acoustical Society of America*, 127(3):1411–1421, 2010.

[15] J. Meaud and K. Grosh. Coupling active hair bundle mechanics, fast adaptation, and somatic motility in a cochlear model. *Biophysical Journal*, 100(11):2576–2585, 2011.

[16] J. Meaud and K. Grosh. Response to a pure tone in a nonlinear mechanical-electrical-acoustical model of the cochlea. *Biophysical Journal*, 102(6):1237–1246, 2012.

[17] J. Meaud and C. Lemons. Nonlinear response to a click in a time-domain model of the mammalian ear. *The Journal of the Acoustical Society of America*, 138(1):193–207, 2015.

[18] H. Motallebzadeh, J. A. Soons, and S. Puria. Cochlear amplification and tuning depend on the cellular arrangement within the organ of corti. *Proceedings of the National Academy of Sciences*, 115(22):5762–5767, 2018.

[19] K. N. O'Connor and S. Puria. Middle-ear circuit model parameters based on a population of human ears. *The Journal of the Acoustical Society of America*, 123(1):197–211, 2008.

[20] E. H. Overstreet, A. N. Temchin, and M. A. Ruggero. Basilar membrane vibrations near the round window of the gerbil cochlea. *JARO-Journal of the Association for Research in Otolaryngology*, 3(3):351–361, 2002.

[21] S. Puria. Measurements of human middle ear forward and reverse acoustics: implications for otoacoustic emissions. *The Journal of the Acoustical Society of America*, 113(5):2773–2789, 2003.

[22] S. Ramamoorthy, N. V. Deo, and K. Grosh. A mechano-electro-acoustical model for the cochlea: response to acoustic stimuli. *The Journal of the Acoustical Society of America*, 121(5):2758–2773, 2007.

[23] T. Ren and A. L. Nuttall. Basilar membrane vibration in the basal turn of the sensitive gerbil cochlea. *Hearing Research*, 151(1):48–60, 2001.

[24] C.-P. Richter, R. Edge, D. Z. He, and P. Dallos. Development of the gerbil inner ear observed in the hemicochlea. *JARO-Journal of the Association for Research in Otolaryngology*, 1(3):195–210, 2000.

[25] C.-P. Richter, G. Emadi, G. Getnick, A. Quesnel, and P. Dallos. Tectorial membrane stiffness gradients. *Biophysical Journal*, 93(6):2265–2276, 2007.

[26] I. J. Russell, P. K. Legan, V. A. Lukashkina, A. N. Lukashkin, R. J. Goodyear, and G. P. Richardson. Sharpened cochlear tuning in a mouse with a genetically modified tectorial membrane. *Nature Neuroscience*, 10(2):215, 2007.

[27] C. A. Shera. Mammalian spontaneous otoacoustic emissions are amplitude-stabilized cochlear standing waves. *The Journal of the Acoustical Society of America*, 114(1):244–262, July 2003.

[28] C. A. Shera and G. Zweig. Reflection of retrograde waves within the cochlea and at the stapes. *The Journal of the Acoustical Society of America*, 89(3):1290–1305, 1991.

[29] D. Strelioff. A computer simulation of the generation and distribution of cochlear potentials. *The Journal of the Acoustical Society of America*, 54(3):620–629, 1973.

[30] Y.-J. Yoon, S. Puria, and C. R. Steele. Intracochlear pressure and derived quantities from a three-dimensional model. *The Journal of the Acoustical Society of America*, 122(2):952–966, 2007.
